# Orphan GPCRs in Neurodegenerative Disorders: Integrating Structural Biology and Drug Discovery Approaches

**DOI:** 10.3390/cimb46100691

**Published:** 2024-10-19

**Authors:** Jinuk Kim, Chulwon Choi

**Affiliations:** Department of Biological Sciences, Seoul National University, Seoul 08826, Republic of Korea; kzzdkssud@snu.ac.kr

**Keywords:** orphan GPCRs, neurodegenerative disorders, Alzheimer’s disease, Parkinson’s disease, structural biology, drug discovery

## Abstract

Neurodegenerative disorders, particularly Alzheimer’s and Parkinson’s diseases, continue to challenge modern medicine despite therapeutic advances. Orphan G-protein-coupled receptors (GPCRs) have emerged as promising targets in the central nervous system, offering new avenues for drug development. This review focuses on the structural biology of orphan GPCRs implicated in these disorders, providing a comprehensive analysis of their molecular architecture and functional mechanisms. We examine recent breakthroughs in structural determination techniques, such as cryo-electron microscopy and X-ray crystallography, which have elucidated the intricate conformations of these receptors. The review highlights how structural insights inform our understanding of orphan GPCR activation, ligand binding and signaling pathways. By integrating structural data with molecular pharmacology, we explore the potential of structure-guided approaches in developing targeted therapeutics toward orphan GPCRs. This structural-biology-centered perspective aims to deepen our comprehension of orphan GPCRs and guide future drug discovery efforts in neurodegenerative disorders.

## 1. Introduction

Neurodegenerative disorders, including Alzheimer’s disease (AD) and Parkinson’s disease (PD), represent a growing global health concern with limited effective treatments [1,2]. The intricate pathophysiology of these conditions involves multiple signaling pathways, many of which are modulated by G-protein-coupled receptors (GPCRs) [3,4]. These receptors play crucial roles in various neuronal functions, including synaptic plasticity [5,6] and neurotransmitter release [7], making them important targets for understanding and potentially treating neurodegenerative diseases [8,9].

The human genome encodes an extensive array of GPCRs, comprising over 800 members, which consist of the largest and most diverse superfamily of membrane proteins [10]. These versatile signaling receptors play crucial roles in numerous physiological processes, responding to a wide range of stimuli from neurotransmitters and hormones to environmental cues [11,12,13]. The significance of GPCRs in human health is underscored by their status as targets for approximately 34% of all FDA-approved drugs, highlighting their immense therapeutic potential [14].

The hallmark of GPCRs is their unique architecture: seven transmembrane α-helices interconnected by intracellular and extracellular loops [15]. This structural arrangement facilitates ligand-induced conformational changes, enabling signal transduction across cell membranes [16]. Within the vast GPCR family, a subset of over 100 receptors remain classified as “orphan” GPCRs. These enigmatic proteins lack identified endogenous ligands [16], posing unique challenges in understanding their activation mechanisms and physiological roles [17]. Despite these obstacles, orphan GPCRs are known to perform crucial functions in various biological processes [18]. Their study has gained significant traction, particularly due to their involvement in central nervous system (CNS) pathologies [19].

The potential of GPCR-targeted therapies, including those targeting orphan GPCRs, is exemplified by recent successes in the pharmaceutical industry [20]. Structure-based drug design has played a crucial role in these advancements. For instance, Novo Nordisk has achieved remarkable success with its GLP-1 receptor-targeting obesity drug, Saxenda, propelling the company to become one of the largest by market capitalization in Europe [21]. In the context of neurodegenerative disorders, orphan GPCRs have emerged as promising novel targets for drug development [22]. Their unexplored nature offers the potential for innovative therapeutic strategies that may yield treatments with improved efficacy and reduced side effects compared to conventional approaches.

This review aims to explore the role of orphan GPCRs in neurodegenerative disorders, with a particular emphasis on recent structural biology insights and their implications for drug discovery. We will examine key orphan GPCRs implicated in Alzheimer’s disease, Parkinson’s disease and other neurodegenerative diseases, discussing how structural characterization has enhanced our understanding of their functions and potential as therapeutic targets. Additionally, we will evaluate current strategies in structure-based drug design targeting orphan GPCRs, emphasizing both the challenges and opportunities in this rapidly evolving field.

## 2. Orphan GPCRs in Neurodegenerative Disorders

GPCRs play crucial roles in cellular signaling through various mechanisms. In general, these receptors activate G proteins (G_s_, G_q_, G_i/o_ and G_11/12_) upon ligand binding, modulating intracellular messengers, such as cyclic AMP (cAMP) or calcium level [23]. GPCRs can also transduce signals via form β-arrestins, or form homo and heterodimers, adding far more complexity to their signaling capabilities [24,25].

**Figure 1:**
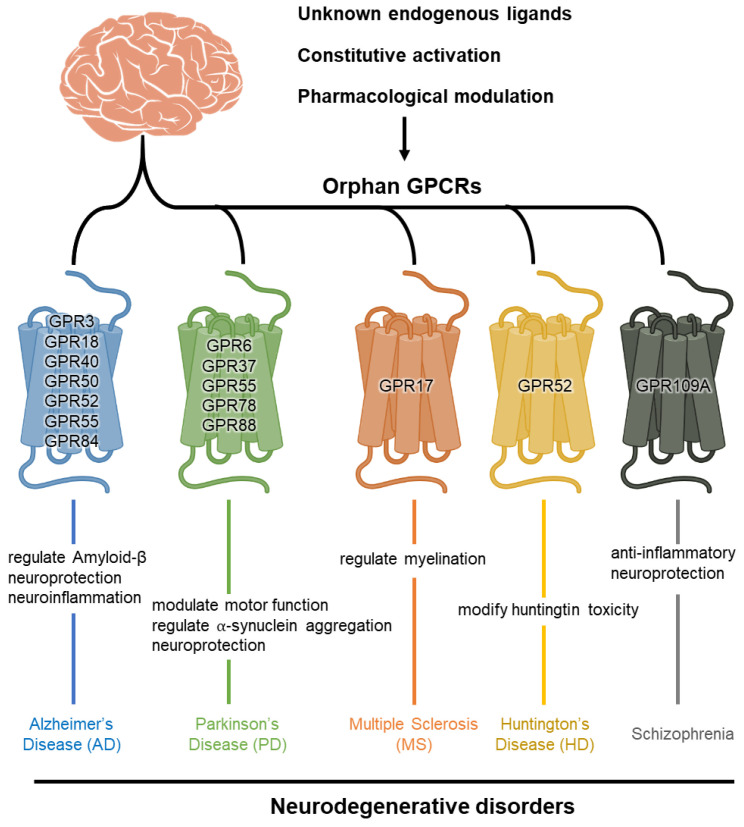
Orphan GPCRs related to neurodegenerative disorders. For representative neurodegenerative disorders, including Alzheimer’s disease (AD), Parkinson’s disease (PD), multiple sclerosis (MS), Huntington’s disease (HD) and schizophrenia, related orphan GPCRs and their brief roles are summarized.

As orphan GPCRs lack identified endogenous ligands, understanding their activation mechanisms presents unique challenges. Although they function similarly to other GPCRs in terms of signaling pathways and mechanisms, the absence of known ligands complicates the study of their roles and therapeutic potential. Nevertheless, their diverse expression patterns in the CNS and potential roles in neuroprotection, neurogenesis and neuroinflammation have made them attractive targets in neurodegenerative disease research (Figure 1).

Recent breakthroughs in structural biology, particularly cryo-electron microscopy (cryo-EM), have revolutionized our understanding of these receptors [26]. These advances have elucidated the inactive and G-protein-bound active conformations of several deorphanized GPCRs, providing crucial insights into their activation mechanisms [27].

In the following sections, we will explore specific orphan GPCRs implicated in various neurodegenerative disorders, focusing on how recent structural insights inform our understanding of their roles in disease pathology and their potential as therapeutic targets. For orphan GPCRs without structural information, a brief overview is provided to acknowledge their significance in neurodegenerative conditions. We place particular emphasis on those with available structural data, allowing for more detailed analysis of their mechanisms and therapeutic relevance. By analyzing these structures, we aim to highlight their therapeutic potential from a structural perspective.

### 2.1. Alzheimer’s Disease

Alzheimer’s disease (AD) is one of the most prevalent neurodegenerative disorders worldwide, characterized by progressive memory loss and cognitive decline [28]. The pathophysiology of AD involves a complex interplay of amyloid-β (Aβ) plaque deposition, tau hyperphosphorylation and neuroinflammation. These processes lead to synaptic dysfunction, neuronal death and brain atrophy, ultimately resulting in dementia and cognitive impairment [28,29,30]. In recent years, several orphan GPCRs have been implicated in various AD-related functions [31,32,33], such as Aβ production, neuroinflammation and neuronal survival, offering new possibilities for therapeutic intervention.

GPR18 is predominantly expressed in immune cells, including microglia, and plays a key role in mediating neuroinflammatory responses [34]. Experimental evidence suggests that GPR18 forms functional complexes with CB2 receptors, influencing microglial activity and potentially reducing neuroinflammation [35]. Activation of GPR18 by resolvin D2 (RvD2) has been shown to alleviate oxidative stress and lower the markers of inflammation in preclinical models, including reductions in amyloid precursor protein (APP) levels [36]. These findings suggest that GPR18 activation may help slow AD progression by mitigating neuroinflammatory damage and providing neuroprotective effects.

GPR40, also known as FFAR1, is expressed in both the pancreas [37] and the brain [38], where it regulates insulin secretion and fatty acid metabolism, respectively. In the CNS, GPR40 has been shown to modulate neuronal activity and influence Aβ production [39]. Evidence from AD mouse models suggests that GPR40 activation improves cognitive function by promoting CREB phosphorylation and elevating the levels of neurotrophic factors, such as BDNF, which support neuroprotection and neurogenesis [40]. These findings make GPR40 a promising target for therapies aimed at addressing neuronal degeneration in AD [41].

GPR50 is predominantly populated in the hypothalamus and is involved in regulating circadian rhythms and energy balance [42,43]. Although its exact endogenous ligand remains unknown, genetic studies have linked GPR50 to an increased risk of AD, particularly in individuals with circadian rhythm disruptions [44,45]. Disturbances in circadian rhythms may exacerbate neurodegenerative processes, such as neuroinflammation and Aβ accumulation, which are the hallmarks of AD [46,47]. GPR50 can form heterodimers with melatonin receptors MT1 and MT2, and its interaction with MT1 interferes with melatonin binding, thereby modulating the melatonin signaling pathway [48,49]. The melatonin pathway plays a role in neuroprotection by regulating oxidative stress and inflammation, processes that are implicated in AD pathology [50]. The ability of GPR50 to influence melatonin signaling suggests its potential impact on AD progression.

GPR55, sometimes referred to as a cannabinoid receptor, is expressed in both the CNS and peripheral tissues [51]. It interacts with the endocannabinoid system, influencing synaptic transmission, inflammation and cell proliferation [52,53]. In the context of AD, GPR55 has been associated with modulating neuroinflammation, potentially reducing microglial activation and preserving synaptic function [54]. GPR55 signaling also affects pathways related to arachidonic acid metabolism, such as the regulation of prostaglandin E2 (PGE2) production in activated microglia, which could further contribute to managing neuroinflammatory responses in AD [53].

#### 2.1.1. GPR3

GPR3 is highly expressed in the CNS and has been implicated in regulating Aβ production and cognitive function in AD models [31]. Overexpression of GPR3 has been shown to increase Aβ secretion, while its deletion reduces Aβ levels, indicating a critical role in AD pathology. Recent structural studies using cryo-electron microscopy (cryo-EM) have provided essential insights into the receptor’s activation mechanisms and ligand-binding properties [55,56,57]. These findings have laid the groundwork for structure-based drug discovery efforts targeting GPR3.

**Figure 2:**
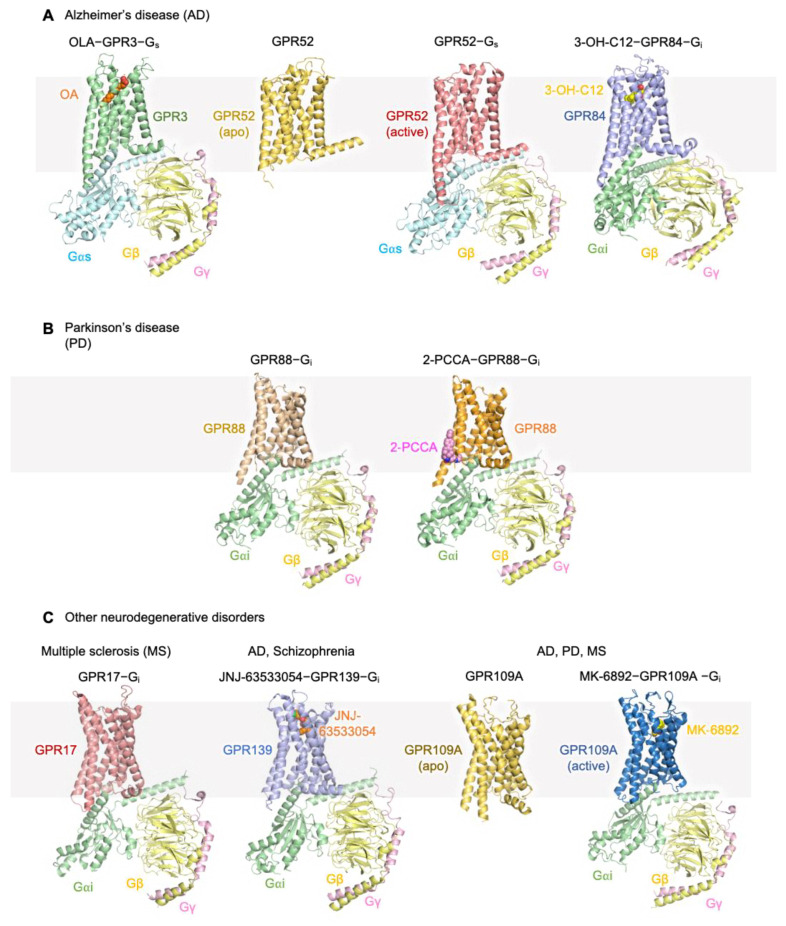
Structures of orphan GPCRs related to neurodegenerative diseases. Reported orphan GPCR structures of AD-related (**A**), PD-related (**B**) and other neurodegenerative diseases (**C**). RCSB PDB IDs of the structures used are 8WW2 (GPR3), 8J18 (GPR84), 6LI1/6LI3 (GPR52), 7WZ4/7EJX (GPR88), 7Y89 (GPR17), 7VUG (GPR139) and 7ZL9/8IHF (GPR109A). For each structure, the GPCR/G protein and ligand are shown as a cartoon and sphere, respectively.

The cryo-EM structure of GPR3 revealed a unique hydrophobic tunnel extending from the extracellular side of the receptor into the membrane bilayer. This tunnel is crucial for the binding of lipophilic ligands, such as oleic acid (OA), which was identified as an endogenous ligand of GPR3. Key residues within the ligand-binding pocket, such as hydrophobic residues in transmembrane helices (TM) 3, 5 and 7, form close contacts with the acyl chain of OA, ensuring stable ligand–receptor interactions. The presence of polar residues at the extracellular entry of the pocket, such as Tyr280 and His96, further anchors the ligand, enhancing receptor activation [55]. The binding of OA stabilizes the receptor in an active conformation, triggering downstream G_s_-mediated signaling pathways. This signaling is critical for regulating neuronal functions and has been linked to both metabolic processes and neuroprotection in AD models (Figure 2A).

Given these structural insights, GPR3 represents a promising target for drug design. The hydrophobic tunnel offers opportunities for the development of small molecules or lipid-based ligands that could modulate GPR3 activity [57]. Structure-guided drug design could focus on enhancing or inhibiting the receptor’s constitutive activity, potentially controlling Aβ production and neuroinflammatory responses in AD. Targeting GPR3 could also lead to the development of therapeutics that mitigate cognitive decline by regulating neuronal signaling and inflammation.

#### 2.1.2. GPR52

GPR52 is highly expressed in the brain, particularly within the striatum, where it plays a key role in modulating dopaminergic signaling and neuroprotection. Its activation has demonstrated neuroprotective effects in cellular models of AD, positioning it as a promising therapeutic target. Recent cryo-EM structures have revealed crucial insights into GPR52′s activation mechanisms and ligand-binding properties [58,59].

One of the most striking features of GPR52 is its high basal activity, meaning the receptor remains active even in the absence of an external ligand (Figure 2A). Structural studies elucidated that this intrinsic activity is driven by a unique extracellular loop 2 (ECL2)**,** which occupies the receptor’s orthosteric binding pocket and functions as a built-in agonist. This structural configuration enables GPR52 to couple with the heterotrimeric G_s_ protein, thereby triggering downstream signaling pathways without an exogenous ligand. The cryo-EM structure has confirmed that this ECL2 “agonist-like motif” (ALM) forms stabilizing interactions with key transmembrane helices, maintaining the receptor in an active conformation [59].

In addition to its orthosteric pocket, GPR52 features a side pocket that can accommodate allosteric ligands. This side pocket presents new opportunities for drug design, particularly for the development of small molecules that can modulate GPR52′s activity by either enhancing or inhibiting its self-activation. The receptor’s constitutive activity, coupled with its ability to modulate intracellular cAMP levels, highlights its potential in regulating neuronal survival and combating neurodegenerative diseases like AD [58].

Furthermore, the apo state structure of GPR52 [58], as determined by X-ray crystallography, provided a detailed view of its inactive conformation. In this state, extracellular loop 2 (ECL2) occupies the orthosteric binding pocket, effectively blocking access of potential external ligands. The apo structure also revealed that transmembrane helix 6 (TM6) remains in an inward-facing position, which is characteristic of inactive GPCRs. This conformation prevents the outward movement necessary for G-protein interaction, thereby keeping the receptor in a pre-activated but not fully functional state.

Given these structural insights, GPR52 represents a highly attractive target for drug discovery [60]. Therapeutic approaches could focus on developing selective modulators that either promote or inhibit its basal activity, potentially offering new strategies for treating AD and other neurodegenerative disorders.

#### 2.1.3. GPR84

GPR84 is predominantly expressed in immune cells, such as monocytes, macrophages and microglia, which are key players in neuroinflammatory processes. In the context of AD, GPR84 activation has been associated with the amplification of inflammatory responses, making it a potential therapeutic target. Recent structural studies using cryo-EM have provided key insights into how GPR84 interacts with ligands and initiates signaling pathways, offering new avenues for drug design aimed at modulating neuroinflammation in AD.

The cryo-EM structure of GPR84 bound to a synthetic agonist LY237 or a potential endogenous ligand, 3-hydroxy lauric acid (3-OH-C12), revealed the receptor’s unique ability to bind medium-chain fatty acids (MCFAs) [61,62]. GPR84′s orthosteric binding pocket is amphipathic, consisting of both hydrophobic and hydrophilic regions that interact with the polar head and hydrophobic tail of ligands, respectively. Notably, the structure showed that extracellular loop 2 (ECL2) adopts a downward-shifted conformation, contributing to ligand binding by forming a roof-like structure above the orthosteric site, effectively sealing the ligand-binding pocket. This downward movement of ECL2 plays a critical role in ligand entry and receptor activation (Figure 2A).

Ligand recognition and activation mechanisms in GPR84 are largely driven by its ability to accommodate lipophilic ligands such as 3-OH-C12. The orthosteric pocket features a unique hydrophobic patch formed by residues F152 and L182, which selectively bind ligands with the appropriate alkyl chain length, thereby defining the specificity for medium-chain fatty acids. Furthermore, interactions between R172 (ECL2) and the polar head of the ligand stabilize the complex and facilitate GPR84 activation. This binding configuration also highlights the receptor’s potential to be selectively modulated by synthetic ligands. Upon activation, GPR84 couples with the G_i_ protein, triggering downstream signaling pathways that enhance the secretion of pro-inflammatory cytokines and promote chemotaxis and phagocytosis in immune cells. This pathway is particularly relevant in AD, where microglial activation exacerbates neuroinflammation. By targeting GPR84, it may be possible to modulate these immune responses and mitigate the inflammatory processes associated with AD progression.

In summary, the structural insights into GPR84′s ligand-binding and activation mechanisms provide a valuable framework for the development of drugs that target neuroinflammatory processes in AD. Modulating GPR84 activity with selective agonists or antagonists could offer novel therapeutic strategies for controlling microglial activation and mitigating the neuroinflammation that contributes to AD progression.

### 2.2. Parkinson’s Disease

Parkinson’s disease (PD) is a chronic neurodegenerative condition marked by the progressive loss of dopaminergic neurons in the substantia nigra, resulting in various motor and non-motor symptoms [63,64]. While traditional therapies have primarily aimed at dopamine replacement, their effectiveness diminishes over time and fails to adequately address non-motor complications, including cognitive impairments and sleep disturbances [65,66]. Additionally, recent research emphasizes the involvement of non-neuronal cells, such as astrocytes and microglia [67], in the progression of PD, highlighting the need to explore therapeutic targets beyond dopamine receptors. The association of several orphan GPCRs with PD pathology has also been reported, suggesting their functional and therapeutic significance.

GPR6 is predominantly expressed in the striatum, where it plays a crucial role in modulating dopamine signaling and motor function through its coupling with Gs proteins to activate the cAMP pathway [68]. Studies in GPR6-knockout animal models have demonstrated that the absence of GPR6 leads to increased dopamine levels in the striatum, resulting in improved motor performance and reduced abnormal movements [69]. These findings suggest that GPR6 antagonists may offer therapeutic potential by modulating dopamine levels more directly, potentially beyond the traditional dopamine replacement therapies used for PD patients. However, further development of more selective and specific GPR6 antagonists, beyond those currently available, such as cannabidiol, is necessary to fully realize its therapeutic potential [70,71].

GPR37, also known as the parkin-associated endothelin-like receptor (PAELR), has been linked to neuroprotection and the regulation of α-synuclein aggregation, a hallmark of PD pathology [72,73,74]. GPR37 activation by its neuropeptide ligand, prosaptide, has been shown to protect dopaminergic neurons from oxidative stress and reduce the aggregation of α-synuclein [75], suggesting GPR37 as an attractive target for developing disease-modifying therapies aimed at preventing neuronal degeneration in PD. Additionally, GPR37′s widespread expression in the brain and CNS [76] enhances its potential as a broad target for neuroprotective strategies.

GPR55 has shown promise in PD research due to its involvement in the endocannabinoid system and its ability to exert neuroprotective effects [77]. Activation of GPR55 by lysophosphatidylinositol (LPI) has been demonstrated to modulate neuroinflammation and promote neuronal survival [78]. In PD models, GPR55 activation was found to reduce neuroinflammatory responses, potentially limiting microglial activation and neurodegeneration [79]. This makes GPR55 a promising therapeutic target for controlling inflammation and supporting neuronal health in PD.

#### GPR88

GPR88, primarily expressed in the striatum, has been implicated in motor control and could be a target for managing PD motor symptoms [80]. GPR88’s ability to modulate GABAergic and glutamatergic signaling positions it as a potential key regulator in PD pathophysiology, as disruptions in these neurotransmitter systems are implicated in the motor and cognitive symptoms of the disease [81].

Recent cryo-EM studies have shed light on the GPR88–G_i1_ signaling complex, revealing critical insights into its activation mechanisms and providing a foundation for the development of structure-based drug designs toward GPR88 [82]. One of the most notable features of GPR88 is its ability to couple with G_i/o_ proteins, a characteristic shared by several GPCRs implicated in neurodegenerative diseases. The cryo-EM structure of GPR88, both in its apo state and in complex with the synthetic agonist 2-PCCA [83], demonstrates that GPR88 exhibits high basal activity, which is thought to contribute to its functional role in the striatum. This high basal activity is mediated through an allosteric binding pocket located on the cytoplasmic side of the receptor, formed by transmembrane segments 5 and 6 (Figure 2B).

In its active state, GPR88’s transmembrane helix 6 (TM6) remains in an inward-facing position, which contrasts with the outward movement typically seen in other GPCRs upon activation. This unique conformation facilitates the binding of G_i_ proteins and enhances downstream signaling without the need for an endogenous ligand, suggesting that GPR88’s activity may be tightly regulated by its environment or through allosteric modulation. Another intriguing feature of GPR88 is the presence of an unidentified electron density in its orthosteric binding site, which likely represents an endogenous ligand, potentially a bioactive lipid. This lipid-like molecule could modulate GPR88’s activity by occupying both the orthosteric and allosteric pockets, thereby offering multiple avenues for drug targeting. Structural studies also highlight the role of cholesterol molecules in stabilizing GPR88’s conformation, suggesting that the lipid environment may play a critical role in GPR88’s activation and signaling pathways [82].

Given its high basal activity and the involvement of its allosteric binding pocket in modulating G_i_ signaling, GPR88 represents a promising drug target for PD. Targeting GPR88 could also provide a novel approach to mitigating dopaminergic dysregulation in PD by modulating GABAergic and glutamatergic transmission, both of which are critically involved in striatal function.

### 2.3. Other Neurodegenerative Disorders

Beyond AD and PD, orphan GPCRs have shown significant relevance in other neurodegenerative disorders. These GPCRs, including GPR26 [84], GPR39 [85] and GPR78 [86], play vital roles in regulating cellular processes, such as neuroinflammation, oligodendrocyte function and neuronal signaling, making them promising targets for therapeutic intervention.

Multiple sclerosis (MS) is a chronic inflammatory disorder characterized by demyelination and neuronal damage within the CNS [87]. In MS, GPR17 has emerged as a key player in regulating oligodendrocyte precursor cell (OPC) differentiation and remyelination [88]. GPR17 has emerged as a key target for therapeutic development in neurodegenerative disorders, where demyelination and the failure to remyelinate are central pathological features [89]. Additionally, GPR55 has shown pro-inflammatory roles in MS models, providing new avenues for targeting neuroinflammation in MS [90].

Huntington’s disease (HD) is a progressive genetic disorder caused by the accumulation of mutant huntingtin (mHTT) protein, leading to motor, cognitive and psychiatric disturbances [91,92]. Among orphan GPCRs, GPR52 plays a significant role in HD by stabilizing mHTT protein levels through cAMP-dependent, PKA-independent mechanisms [93,94]. Inhibition of GPR52 has been shown to reduce mHTT levels and ameliorate HD-like phenotypes in animal models. Furthermore, GPR52 antagonists are being explored as potential therapeutic agents to alleviate HD-related motor symptoms and cognitive deficits [94,95].

#### 2.3.1. GPR17

Recent cryo-EM structural studies have provided key insights into GPR17’s activation mechanisms (Figure 2C). The structure of GPR17 in complex with G_i_ proteins revealed several unique structural features that underlie its function. Notably, extracellular loop 2 (ECL2) occupies the orthosteric binding pocket, a feature that distinguishes GPR17 from other Class A GPCRs. This occupation of the ligand-binding site by ECL2 promotes self-activation, where GPR17 can activate downstream signaling pathways even in the absence of external ligands [96]. The structural insights into GPR17’s self-activation mechanism and G_i_-coupled signaling pathways provide a valuable foundation for the development of structure-based drug designs aimed at modulating GPR17 activity. Targeting the orthosteric binding pocket and the unique ECL2 configuration may allow for the design of selective agonists or antagonists that can either enhance or inhibit GPR17’s function, offering novel therapeutic approaches for MS and other neurodegenerative diseases.

#### 2.3.2. GPR139

GPR139 is an orphan GPCR highly expressed in the CNS, particularly in regions associated with neuroprotection, cognition and neuroinflammation [97]. Although GPR139 is less studied compared to other GPCRs, recent research has revealed its significant role in various neurodegenerative diseases, such as schizophrenia [98] and AD [99]. Its neuroprotective effects in animal models have drawn attention to its potential as a therapeutic target.

Structurally, GPR139 is activated by endogenous amino acids, such as L-tryptophan (L-Trp) and L-phenylalanine (L-Phe). These ligands bind within the orthosteric pocket, interacting with key residues, such as R244 and W241. Recent cryo-EM studies have provided insights into how the synthetic agonist JNJ-63533054 binds to GPR139, stabilizing it in an active conformation. The structural analysis of GPR139 in complex with miniG_s/q_ and G_i_ proteins revealed two distinct ligand-binding poses, suggesting a ligand flexibility that may allow for selective modulation of the receptor based on different physiological conditions [100]. These findings provide a foundation for structure-based drug design targeting GPR139 for neuroprotective therapies (Figure 2C).

Interestingly, GPR139 also forms a heterodimer with the μ-opioid receptor (MOR), where it negatively regulates opioid receptor function, signaling and trafficking. This interaction highlights a unique regulatory mechanism where GPR139 can counterbalance MOR activity, potentially offering a new therapeutic angle for treating opioid addiction and related disorders. This heterodimerization influences MOR’s sensitivity to opioids, including morphine, and has been shown to affect opioid-induced signaling and tolerance development [101].

The combination of GPR139′s involvement in neuroprotective signaling and its ligand flexibility makes it an exciting candidate for therapeutic interventions in neurodegenerative diseases and opioid use disorders. Future studies aimed at developing selective agonists or allosteric modulators of GPR139 could pave the way for novel treatments that address the neuroprotective and regulatory roles of this orphan GPCR.

#### 2.3.3. GPR109A

GPR109A, also known as the niacin receptor or HCA2, is abundant in the cerebral cortex and plays a critical role in regulating lipid metabolism, immune responses [102] and neuroinflammation [103]. Its involvement in both metabolic and inflammatory pathways has made it a promising target for treating various neurodegenerative diseases, such as MS [104], PD [105] and AD [106]. Activation of GPR109A by endogenous ligands like 3-hydroxybutyric acid or pharmacological agents such as niacin reduces neuroinflammation, which could potentially slow the progression of demyelination and support remyelination [107].

Recent structural studies have revealed important details about its activation and signaling mechanisms, further highlighting its therapeutic potential. The cryo-EM structure of the GPR109A–G_i_ complex has been resolved, providing detailed insights into its activation (Figure 2C). The study shows that MK-6892, a potent GPR109A agonist, binds within the orthosteric binding pocket, initiating significant conformational changes. Specifically, the binding of MK-6892 induces movement in transmembrane helices 5 and 6, which are key components of the receptor’s activation mechanism. These structural changes promote the coupling of GPR109A with G_i_ proteins, effectively modulating the intracellular signaling pathways involved in immune response and lipid metabolism [108,109].

In addition, the crystal structure of GPR109A in its inactive state has also been resolved, showing that the receptor is locked in an inactive conformation by specific mutations (S287V), which hinder ligand binding and downstream signaling (Figure 2C). This inactive state provides a comparative framework for understanding the transition between inactive and active states of GPR109A [108].

Given these structural insights, targeting GPR109A with selective modulators holds promise for treating neurodegenerative diseases by mitigating inflammation and supporting neuronal survival. The detailed structural understanding of its ligand-binding properties and signaling mechanisms provides a foundation for future structure-based drug design efforts. Moreover, the receptor’s ability to couple exclusively with G_i/o_ proteins makes it an attractive candidate for developing biased ligands that could minimize side effects, such as niacin-induced flushing, while maximizing its therapeutic effects.

## 3. Drug Development Strategies

The development of drugs targeting orphan GPCRs in neurodegenerative disorders requires an array of strategies, ranging from traditional screening methods to advanced computational approaches. These methodologies offer unique advantages and limitations in navigating the complex landscape of orphan GPCR drug discovery [110]. In this section, we explore high-throughput screening (HTS) and structure–activity relationship (SAR) studies, the more precise structure-based drug design (SBDD) methods and the emerging role of artificial intelligence (AI)-based techniques in orphan GPCR-targeted drug development.

### 3.1. High-Throughput Screening (HTS) and Structure–Activity Relationship (SAR) Studies

High-throughput screening (HTS) is one of the most widely used methods for identifying active compounds that can modulate the function of target receptors [111]. This approach involves testing large chemical libraries against the receptor of interest, allowing for the rapid identification of potential agonists, antagonists or modulators. HTS is particularly advantageous for orphan GPCRs, where the absence of known endogenous ligands often makes it difficult to characterize receptor activity [112]. Screening vast libraries can therefore provide initial lead compounds for further development toward orphan GPCRs.

**Figure 3:**
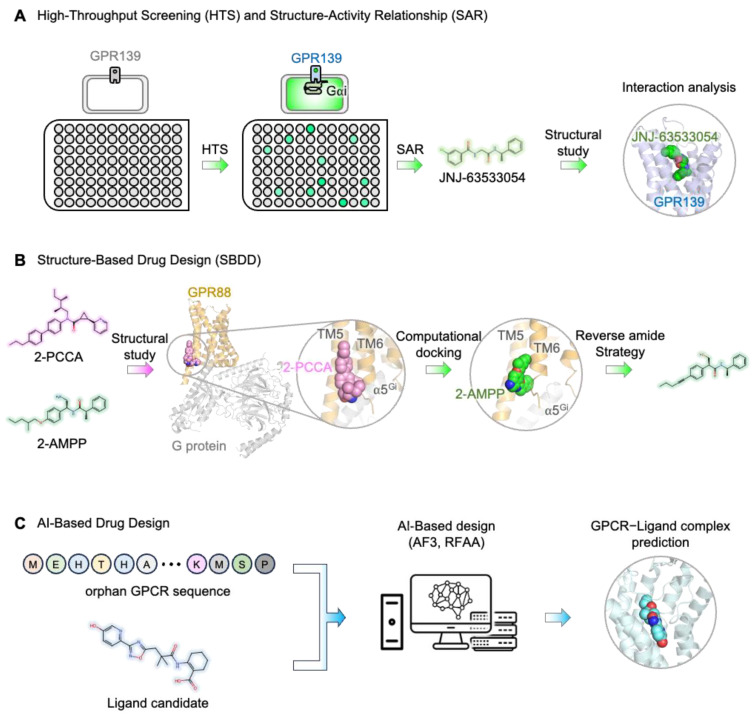
Drug development strategies targeting orphan GPCRs. (**A**) Schematic representation of the high-throughput screening (HTS) and structure–activity relationship (SAR) approach focused on the case of drug discovery for GPR139. (**B**) Schematic representation of the structure-based drug design (SBDD) for GPR88-targeting drug discovery. (**C**) Brief scheme of AI-based drug design.

An illustrative example is GPR139, where a focused HTS of a 100K compound library led to the identification of initial hits, which were subsequently refined through structure–activity relationship (SAR) studies [113] (Figure 3A). This effort culminated in the discovery of (S)-3-chloro-N-(2-oxo-2-((1-phenylethyl)amino)ethyl)benzamide, known as JNJ-63533054, a selective and potent agonist with an EC50 of 16 nM. The compound exhibited favorable drug-like properties, including the ability to cross the blood–brain barrier, making it a strong candidate for treating conditions such as schizophrenia [113]. Following HTS, SAR studies were crucial in modifying the chemical structure of these hits to enhance receptor binding and activity. In the case of GPR139, SAR analysis involved varying the linker region and substituents on the aryl benzamide moiety, with findings indicating that the 3-chloro substituent significantly improved binding affinity and selectivity. Structural studies revealed the active conformation of GPR139 when bound to JNJ-63533054, providing molecular insights into how this drug candidate works [100] (Figure 2C).

Similarly, the development of TAK-041, another potent GPR139 agonist, employed a combination of HTS and medicinal chemistry optimization [114,115]. TAK-041, also known as NBI-1065846, was discovered through a medicinal chemistry strategy that identified benzotriazinone-based agonists with selective activity. It showed efficacy in modulating the habenula circuit in vivo [114], which plays a role in psychiatric conditions, such as schizophrenia. Further pharmacological studies demonstrated that TAK-041 could improve social behavior deficits in animal models. This example underscores the potential of HTS in conjunction with SAR to develop compounds with targeted receptor activity.

While HTS and SAR provide a powerful toolkit for drug discovery, they come with limitations, including significant time and resource investment. To streamline this process, many drug discovery programs now employ virtual screening as an initial step. This technique leverages computational methods to rapidly filter large compound libraries, identifying the most promising candidates for further experimental testing [116]. By focusing the HTS on these pre-screened compounds, researchers can reduce costs and time while increasing the likelihood of discovering viable hits. Virtual screening is especially valuable for orphan GPCRs, where endogenous ligands are unknown, as it can predict potential ligand–receptor interactions.

### 3.2. Structure-Based Drug Design (SBDD)

Structure-based drug design (SBDD) has become a cornerstone of GPCR-targeted drug development, offering a more rational and targeted approach by leveraging high-resolution structural information of receptors [117]. For orphan GPCRs, SBDD provides a strategic advantage in the design of ligands that fit precisely within the receptor’s binding pockets. Recent advancements in cryo-EM and X-ray crystallography have facilitated the determination of several orphan GPCR structures, providing valuable insights for virtual screening and ligand optimization.

An example of SBDD’s application in orphan GPCRs is GPR88 (Figure 3B). Following the elucidation of its structure through cryo-EM, researchers investigated the binding interactions of 2-PCCA, a known agonist of GPR88 (Figure 2B). The structural insights obtained from the 2-PCCA–GPR88 complex revealed that 2-PCCA binds to an allosteric site formed by transmembrane segments TM5 and TM6 and the α5 helix of the G_i_ protein [83]. With this information, researchers employed a “reversed amide” strategy to design a new scaffold for potential agonists. A series of (4-substituted-phenyl)acetamides were synthesized and tested, leading to the discovery of novel compounds that exhibited better or comparable potency to 2-AMPP, another known GPR88 agonist [118]. Computational docking studies further confirmed that these new compounds occupy the same allosteric site on GPR88 as 2-PCCA, demonstrating how SBDD can guide the rational design of improved therapeutics.

Despite its strengths, SBDD is not without limitations. For orphan GPCRs with unknown structures, direct application of SBDD is impossible. Obtaining high-resolution structures of GPCRs via X-ray crystallography and cryo-EM presents challenges, including difficulties in expressing and purifying functional receptors. X-ray crystallography is limited by the flexible regions and dynamic nature of GPCR, which make crystallization challenging. Cryo-EM does not require crystallization but still faces challenges with orphan GPCRs. Stabilizing the receptor in an active state, in complex with a G protein, is difficult without a known ligand, and resolving the inactive state remains challenging due to the small size of the receptor [119]. In such cases, researchers turn to homology modeling and structure prediction to generate receptor models, although these often lack the accuracy required for high-quality drug design [120].

### 3.3. AI-Based Approaches in Drug Design

The recent advancements in artificial intelligence (AI) technologies have opened new frontiers in drug design, particularly for orphan GPCRs. Deep-learning-based models, such as AlphaFold2 [121] and RoseTTAFold2 [122], have demonstrated remarkable capabilities in accurately predicting the three-dimensional (3D) structures of proteins, including orphan GPCRs (Figure 3C). These predictive models allow researchers to generate structural information that was previously challenging to obtain, offering an opportunity to use virtual screening methods to identify potential drug candidates.

AlphaFold2 in particular has shown promise in virtual drug screening for GPCRs. In a study focusing on Class A GPCRs, AlphaFold2-generated models were capable of predicting ligand-binding poses with an accuracy close to that of experimentally determined structures [123]. This capability suggests that, even when experimental structural data for an orphan GPCR are lacking, AI-based models can predict a reliable receptor structure, thus facilitating virtual screening. By using molecular docking, researchers can rapidly narrow down vast chemical libraries to identify promising candidates, significantly reducing the time and cost associated with traditional HTS and SAR studies [124]. Recent efforts have utilized AlphaFold2-predicted structures of orphan GPCRs implicated in neurodegenerative diseases, such as Alzheimer’s disease, to guide virtual screening campaigns and successfully identify novel potential ligands.

Another exciting development in AI-based drug design is the use of multitask learning models. For instance, a recent study employed a multitask model to predict the bioactivity of chemical compounds against various GPCRs, including orphan receptors [125]. By incorporating both protein sequence features and ligand information, the model can transfer knowledge from well-characterized GPCRs to those with less available data. This approach not only facilitates bioactivity prediction but also identifies potential ligand–receptor interactions, thereby guiding drug design even in the absence of detailed 3D structures [126].

In addition to these advances, AI-driven generative models are pioneering a new era in drug design. Diffusion-based models, such as those used in AlphaFold3 [127] and RoseTTAFold All-Atom [128], offer the potential to design both proteins and ligands directly. This capability allows for the creation of entirely new compounds that are tailored to fit the predicted binding pockets of orphan GPCRs. Although direct applications of diffusion-based drug designs for orphan GPCRs are still emerging, these technologies hold immense promise. They can explore the chemical space beyond traditional databases, potentially discovering novel ligands with high specificity and efficacy. However, due to complex interactions and conformational changes that can affect ligand behavior and efficacy, predicting ligand efficiency solely with AI remains challenging. Therefore, AI-based predictions should be complemented with HTS to experimentally validate candidate compounds, ensuring their efficacy in biological systems.

In summary, AI-based approaches are reshaping the landscape of orphan GPCR drug discovery. From AlphaFold2 and RoseTTAFold facilitating structure prediction and virtual screening to multitask learning models and diffusion-based generative methods driving the design of new ligands, these technologies provide a comprehensive toolkit. Leveraging these AI advancements enables researchers to efficiently target orphan GPCRs, ultimately accelerating the development of therapeutics for various neurodegenerative diseases.

## 4. Discussion and Future Perspectives

The exploration of orphan GPCRs in neurodegenerative disorders represents a promising frontier in drug discovery. This review highlighted the diverse strategies employed to tackle the complexities of these receptors and harness their therapeutic potential. The integration of structural biology, computational methods and innovative drug design techniques has facilitated the targeting of orphan GPCRs implicated in conditions like Alzheimer’s and Parkinson’s diseases.

The structural elucidation of receptors such as GPR52, GPR88 and GPR17 has provided crucial insights into their functions and ligand interactions, greatly enhancing the drug design process. The success of structure-based drug design (SBDD) in identifying novel ligands underscores the importance of continued investment in cryo-EM and X-ray crystallography research. Furthermore, high-throughput screening (HTS) and SAR studies, when complemented by virtual screening, offer a powerful approach to rapidly identify and optimize potential compounds, particularly in the challenging landscape of orphan receptor drug discovery.

The rise of AI-based technologies, including AlphaFold2 and RoseTTAFold, has further revolutionized the field by providing accurate structural prediction for orphan GPCRs. These tools facilitate virtual screening and open up possibilities for multitask learning models and diffusion-based generative methods to design new ligands. The combination of these advanced computational methods with traditional drug discovery processes has accelerated the identification of lead compounds and provided new directions for therapeutic development.

Despite recent advancements, challenges remain in orphan GPCR drug discovery. The absence of known endogenous ligands complicates compound identification and validation, and the complex signaling pathways in neurodegenerative disorders require careful consideration to minimize off-target effects. Moving forward, refining AI-powered tools to predict dynamic receptor–ligand interactions, exploring allosteric modulation for greater selectivity and integrating multi-omics data with structural studies could significantly enhance our understanding of orphan GPCRs and guide therapeutic development. The convergence of structural biology, computational methods and innovative drug design holds great potential for creating transformative treatments that could profoundly impact those affected by these debilitating conditions.

## Data Availability

Not applicable.
